# High Sensitivity Electrochemical Cholesterol Sensor Utilizing a Vertically Aligned Carbon Nanotube Electrode with Electropolymerized Enzyme Immobilization

**DOI:** 10.3390/s91108658

**Published:** 2009-10-29

**Authors:** Anurat Wisitsoraat, Chanpen Karuwan, Krongkamol Wong-ek, Ditsayut Phokharatkul, Pornpimol Sritongkham, Adisorn Tuantranont

**Affiliations:** Nanoelectronics and MEMS Laboratory, National Electronics and Computer Technology Center, 112 Thailand Science Park, Pahol Yothin Rd., Klong Luang, Pathumthani 12120, Thailand; E-Mails: chanpen.karuwan@nectec.or.th (C.K.); wongek@tu.ac.th (K.W.); ditsayut.phokharatkul@nectec.or.th (D.P.); pornpimol.sritongkham@nectec.or.th (P.S.); adisorn.tuantranont@nectec.or.th (A.T.)

**Keywords:** vertically aligned varbon nanotubes, cholesterol, polyaniline, electropolymerization, enzyme based biosensor

## Abstract

In this report, a new cholesterol sensor is developed based on a vertically aligned CNT electrode with two-step electrochemical polymerized enzyme immobilization. Vertically aligned CNTs are selectively grown on a 1 mm^2^ window of gold coated SiO_2_/Si substrate by thermal chemical vapor deposition (CVD) with gravity effect and water-assisted etching. CNTs are then simultaneously functionalized and enzyme immobilized by electrochemical polymerization of polyaniline and cholesterol enzymes. Subsequently, ineffective enzymes are removed and new enzymes are electrochemically recharged. Scanning electron microscopic characterization indicates polymer-enzyme nanoparticle coating on CNT surface. Cyclic voltammogram (CV) measurements in cholesterol solution show the oxidation and reduction peaks centered around 450 and −220 mV, respectively. An approximately linear relationship between the cholesterol concentration and the response current could be observed in the concentration range of 50–300 mg/dl with a sensitivity of approximately 0.22 μA/mg·dl^−1^, which is considerably higher compared to previously reported CNT bioprobe. In addition, good specificity toward glucose, uric acid acetaminophen and ascorbic acid have been obtained. Moreover, sensors have satisfactory stability, repeatability and life time. Therefore, the electropolymerized CNT bioprobe is promising for cholesterol detection in normal cholesterol concentration in human blood.

## 1. Introduction

Cholesterol is an essential lipid for human body. The desired total plasma cholesterol for an individual is less than 5.2 mM (200 mg/dL) and it poses a potential health threat when the level is greater than 6.2 mM (240 mg/dL). Excessive plasma cholesterol causes poor cardiovascular conditions, such as atherosclerosis and hypertension, which can lead to coronary heart disease, myocardial and cerebral infarction. The total cholesterol in all lipoprotein fractions is normally determined using a colorimetric assay specific for cholesterol [1]. In the process, cholesterol esters are acted upon by cholesterol esterase (ChEs) to release free cholesterol and then cholesterol oxidase (ChOx) catalyzes the reaction to generate H_2_O_2_, which is reacted to yield quinoneimine dye. The absorbance of the dye is proportional to H_2_O_2_, hence the cholesterol concentration. The disadvantages of this technique include instability of color reagent, variable reactivity of ester, corrosive nature of reagents, poor specificity and unreusable reagents.

Electrochemical detection techniques emulate the colorimetric assay by immobilizing ChEs and ChOx onto electrode surfaces to liberate cholesterol and subsequently generate H_2_O_2_, which is measured amperometrically [2]. Its advantages include rapid analysis, reusability, thermal stability, linearity and good specificity. However, electrochemical biosensors still need further development to improve performances in term of sensitivity, reproducibility and life time. Several approaches including functionalization of the working electrode surface by polymer or nanomaterials have been proposed to enhance the performance of electrochemical biosensors [3,4].

Carbon nanotubes (CNTs) have been shown to promote electron transfer reactions for the redox of important biomolecules [5-8]. In addition, vertical alignment of the CNT nanoeletrodes is preferred over other schemes because the open end of a CNT is expected to show faster electron transfer rate (ETR). This characteristic along with their high surface area and conformal compatibility with biomolecules makes CNTs particularly attractive for high-sensitivity biochemical sensing systems. However, the performance of CNTs based electrochemical biosensor is also critically dependant on effectiveness of enzyme immobilization on CNT structure. Conventional approaches for enzyme immobilization on amperometric bioprobes include direct adsorption [9] and entrapment in polymeric films [10]. Physical adsorption technique suffers from protein desorption due to changes in temperature, pH and ionic strength while the enzyme entrapment in the lattice of a polymer matrix or membrane provides relatively better enzyme retention.

Recently, vertically aligned CNT electrode with enzyme entrapment in polyvinyl alcohol (PVA) by spin coating has been reported [8]. Nevertheless, the disadvantages of the reported technique are low enzyme entrapment efficiency and life-time due to PVA's low physical entrapment capacity, low conductivity and partial water solubility. Electrochemical enzyme entrapment with conducting polymer should offer much higher enzyme retention capacity and better electron transfer to CNTs [12-16]. There have been some reports on biosensors based on CNTs-enzyme-polymer composites prepared by electrochemical polymerization [17-22]. However, there have been few reports that perform enzyme immobilization by electrochemical polymerization on vertically aligned CNTs electrode, which can offer significantly improved sensor's performance and reliability. In this work, cholesterol bioprobe is developed based on vertically aligned CNTs with enzyme immobilization in polyaniline (PANI) using two-step electrochemical process.

## 2. Experimental Section

**Figure 1.**
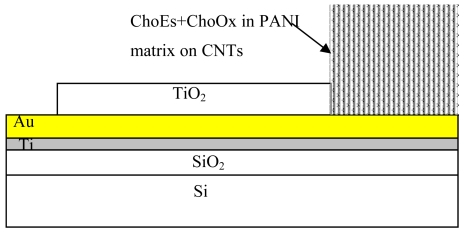
Structure of the CNT based cholesterol bioprobe.

The structure for electrode fabrication is shown in Figure 1. First, SiO_2_ (400 nm), Cr (50 nm) and Au (500 nm) were successively sputtered on <100> Si substrates. Next, aluminium oxide (10 nm) and stainless steel (SS) catalyst (5 nm) were sequentially sputtered to prepare for CNT synthesis. Titanium dioxide was then sputtered via shadow masking on the gold layer over a defined region, which excludes active sensing area (1 mm^2^) and electrical contact region. The aluminium oxide and titanium dioxide layers were deposited by reactive sputtering at a pressure of 3 × 10^−3^ mbar of 1:5 Ar/O_2_ gas mixtures while other metallic layers were deposited in Ar gas at the same pressure.

Vertically aligned carbon nanotubes were grown by thermal chemical vapor deposition (CVD) with gravity effect and water-assisted etching. The catalyst layers on substrates were placed upside down along gravitational field on an alumina carrier in a horizontal furnace thermal CVD system. The CNT synthesis was conducted at atmospheric pressure and growth temperature of 700 °C. During CNT growth, acetylene was flown for 1.5 minute and hydrogen to acetylene volume flow ratio was 4.3:1 with hydrogen flow of 1,935 sccm and acetylene flow of 450 sccm. In the course of CNT growth, in-situ-water-assisted etching was employed to remove undesired amorphous carbon formation from random acetylene decomposition. In water etching process, 300 ppm of water vapor was introduced by water bubbling through Ar gas for 3 minutes while acetylene gas was turned off. CNTs growth and water-assisted etching were repeatedly performed for three cycles and the total time was 13.5 minutes. CNTs' functionalization and enzyme immobilization in PANI matrix were then conducted as per the following protocol.

### Reagents for biosensing

Analytical-grade bio-reagents used in this study: cholesterol (95%), cholesterol oxidase (ChOx, *Pseudomonas fluorescens* 25 U/mg), cholesterol esterase (ChEs, *Pseudomonas fluorescens* 100 U/mg), horseradish peroxidase (HRP, 5,000 U), potassium ferrocyanide, trehalose, Triton X-100 were purchased from Sigma-Aldrich. Aniline monomer and phosphate buffer saline (PBS) solution was purchased from Fluka (USA).

### Surface functionalization and enzyme immobilization

Functionalization of the CNT based electrode was done by electrochemical polymerization on the active zone of the working electrode in a solution of 0.2 M aniline monomer. Prior to aniline polymerization, CNT was refluxed in 1 M nitric acid for 2 hours at room temperature to remove catalyst and clean CNT surface. The aniline solution was prepared by dissolving 4.8 mL of aniline monomer and 1 mL of HCl in 100 mL of PBS solution (pH 7.0) with constant stirring for 10 minutes at room temperature. The final pH of aniline solution is around 2.5. Electrochemical polymerization was performed at ∼ 20 °C by a standard three electrode electrochemical system (Metrohm electrochemical workstation with BAS C3 voltammetric cell). The working electrode (WE) was the CNT based electrode while the counter electrode (CE) was a Pt wire and all the potentials were controlled and measured as referred to an Ag|AgCl|KCl (sat.) reference electrode (RE). During polymerization, the CNT electrode was biased at a constant voltage of 0.6 V with respect to RE for 30 minutes. The polyaniline matrix formed on CNTs introduced NH group on CNT surface, which greatly helped immobilizing various biological analytes, particularly proteins [12,13].

Enzyme immobilization was then followed by a two-step electrochemical procedure. The first electrochemical process was conducted in the mixture of 2 mL of cholesterol enzyme and 2 mL of 0.2 M aniline monomer. The pH of the mixture was measured to be about 3. The cholesterol enzyme was prepared by dissolving 1.5 mg ChEs, 2.0 mg ChOx, 1.8 mg HRP, 8.0 mg potassium ferrocyanide (redox mediator) and 6.0 mg trehalose (enzyme stabilizer) in 0.3 mL PBS solution. The PANI-CNT electrode was biased at a constant voltage of 0.6 V with respect to RE for 30 minutes. Due to the acidic condition during aniline polymerization, cholesterol enzymes were inactive or even denatured. Thus, ineffective enzymes were removed by etching in stirred 6 M HCl solution for 2 hours, leaving cavities in and on PANI matrix. The PANI layer was then reduced by biasing at constant voltage of −0.2 V for 20 minutes in PBS solution to remove any residual anions from acidic etching. New enzymes were electrochemically recharged into PANI matrix by biasing the electrode in cholesterol enzyme in PBS solution (pH 6.0) at a constant voltage of 0.6 V with respect to RE for 30 minutes. After immobilization, the sensor chip was stored in a refrigerator for 24 h and thoroughly rinsed in PBS before running each test.

### Electrochemical measurements

Electrochemical measurement of cholesterol was performed by the same electrochemical system. Due to cholesterol's water insolubility, the stock cholesterol solution was prepared by dissolving 40 mg cholesterol in 1 mL Triton X-100 and 9 mL 0.1M PBS solution (pH 7.0) and stirring with a magnetic spin bar just before the measurement to obtain a homogeneous solution. Cholesterol solution with different concentration was then prepared for testing by proper dilution of the stock solution. Cholesterol bioprobe was immersed in 0.1 M PBS solution and cyclic voltammograms (CV) were run at several scan rates for different concentrations of cholesterol. The voltage window for CV was from −1.0 to +1.0 V because the redox peaks appeared within this potential window.

## 3. Results and Discussion

**Figure 2.**
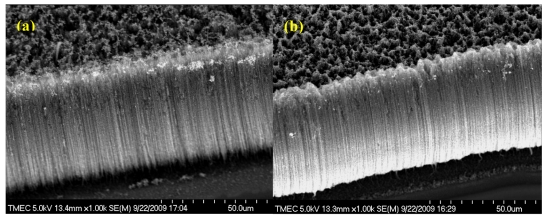
Typical SEM micrograph of CNT electrode (a) before and (b) after electrochemical polyaniline-enzyme coating.

**Figure 3.**
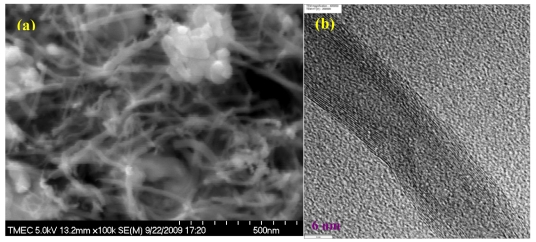
High magnification (a) top view SEM image of CNT electrodes after electrochemical polyaniline-enzyme coating and (b) TEM image of CNT extracted from the synthesized vertically aligned CNTs.

The surface morphology of the sensing area of CNT based biochip was examined using scanning electron microscopy (SEM). Scanning electron microscopic images of vertically aligned CNTs before and after polyaniline-enzyme immobilization,is shown in Figures 2(a) and (b), respectively. The cross section seen in the images confirms that CNTs are indeed vertically aligned, with a vertical height of ∼50 μm. In addition, it can be seen that vertically aligned CNTs are uniformly coated by polymer-enzyme nanoparticles on CNT surface. Figure 3(a) shows high magnification top view SEM image of CNT electrodes after electrochemical polyaniline-enzyme coating. It can be seen the size of polyaniline-enzyme nanoparticles is in the range between 20 and 50 nm. In addition, transmission electron microscopic (TEM) characterization as shown in Figure 3(b) verifies that CNTs are multiwalls with diameters and numbers of walls in the range of 10–25 nm and 8–15 respectively.

**Figure 4.**
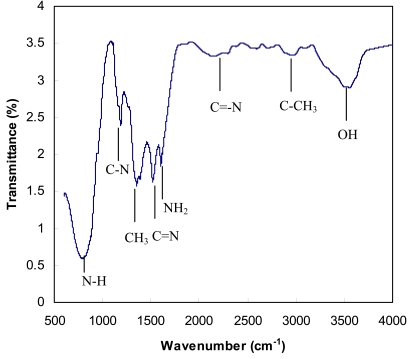
Typical FT-IR spectrum of the CNT electrode after electrochemical polyaniline-enzyme coating.

The chemical property of the CNT bioprobe was characterized by Fourier Transform Infrared Spectroscopy (FTIR) using a Perkin Elmer System—2000R FTIR spectrophotometer. The FTIR spectrum of the CNT bioprobe is shown in Figure 4. The observed functional groups confirm successful polyaniline formation and cholesterol enzyme immobilization. The PANI, ChOx, and ChEs exhibit common characteristic bands at around 2,852, 1,565, and 1,300 cm^−1^, which are the C–H bending vibration out of the plane of the para-disubstituted benzene rings, the C–C stretching band in benzenoid rings, the C–N stretching mode. In addition, PANI have distinctive peaks at 1,143 and 796 cm^−1^, the vibration band of the anion (HCl–PAni), and N–H vibration band of aniline, respectively. Likewise, ChOx and ChEs have distinct absorption peaks at approximately 1,424, 2,867 and 3,524 cm^−1^, which correspond to the CH_3_, C–CH_3_, OH vibration bands.

**Figure 5.**
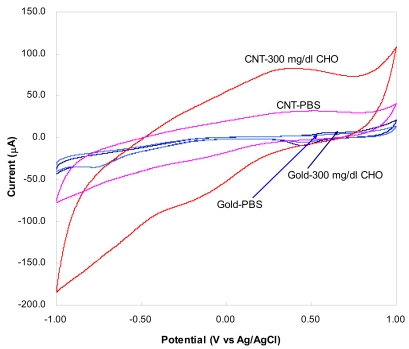
Cyclic voltammograms for gold and CNT based cholesterol bioprobe at 300 mg/dL concentration and 100 mV scan rates coating.

CV curves for gold based and CNT based cholesterol bioprobe at a cholesterol concentration of 300 mg·dL^−1^ and 100 mV scan rate are shown in Figure 5. The gold based bioprobe was prepared by the same two-step electrochemical procedure as that was done for CNT based bioprobe. It is evident that CNT based bioprobe show much higher current sensitivity than gold based cholesterol bioprobe. The result confirms the enhancement of electrochemical detection of cholesterol by vertically aligned CNT structures. However, this result is in contrast to Lee's report [22] that Pd sputtered screen-printed carbon electrodes (SPCEs) exhibited higher electrochemical sensitivity than CNT modified SPCEs. The contradiction can be explained from the fact that the substrate surfaces for sputtered thin film coating are significantly different.

In Lee's work, screen-printed carbon electrodes (SPCEs) were used but standard SiO_2_/Si substrates were utilized in this work. The surfaces of SPCEs were very rough and pitted while SiO_2_/Si substrates were very smooth. The Pd sputtering caused better enhancement than CNTs modification on SPCEs because thin Pd sputtering could directly increase electron transfer rate without losing surface area whereas CNTs modification would reduce electrode surface area as Nafion/CNTs filled into pores of SPCE surface. In our work, vertically aligned CNTs that were directly grown on gold sputtered SiO_2_/Si electrode significantly increased specific surface area and electron transfer rate.

From Figure 5, the anodic and the cathodic peaks at scan rate of 0.1 V/s are centered on 397 and 526 mV, respectively. This could be ascribed to the electrocatalytic reduction of H_2_O_2_ by horseradish peroxidase following cholesterol release and decomposition by ChEs and ChOx, respectively [11], as illustrated in the following set of reactions:

(1) cholesterol ester→fatty acid+cholesterol(cholesterol esterase)

(2) $$\text{cholesterol}\to \text{cholest}-4-\text{ene}-3\text{one}+{\text{H}}_{2}{\text{O}}_{2}\;\left(\text{cholesterol oxidase}\right)$$

(3) $${\text{H}}_{2}{\text{O}}_{2}+2{\left[\text{Fe}{\left(\text{CN}\right)}_{6}\right]}^{4-}+{2\text{H}}^{+}-\to 2{\left[\text{Fe}{\left(\text{CN}\right)}_{6}\right]}^{3-}+{2\text{H}}_{2}\text{O}\;\left(\text{horseradish peroxidase}\right)$$

**Figure 6.**
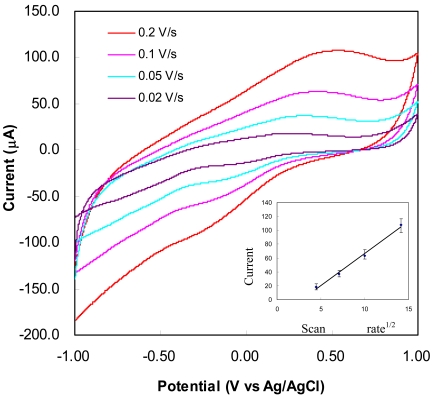
Cyclic voltammograms for cholesterol bioprobe at 300 mg/dL concentration with different scan rates. Inset: Peak current (oxidation peaks) as a function of the square root of scan rate. The error bars indicates the variation from five independent measurements.

CV curves for cholesterol bioprobe at a cholesterol concentration of 300 mg.dl^−1^ with different scan rates are shown in Figure 6. It can be noticed from the given set of CV curves that magnitude of the anodic and cathodic peaks are different for different scan rates. The anodic peaks shift from 490 mV to 540 mV as the scan rate increases from 10 mV/s to 300 mV/s while the cathodic peaks change from −250 mV to −333 mV. Moreover, it can be seen that the redox reaction is not reversible since the peak current ratio between anodic and cathodic (i_pa_/i_pc_) is not equal to one for all scan rates. This implies that the electron transfer rate in the chemical reactions, which are coupled to the redox process or to the adsorption of either reactants or products that might occur during the scanning process, is slow relative to the voltage scan rate. Furthermore, the oxidation peak amplitude is found to increase linearly with the square root of scan rate as shown in the inset of Figure 6, indicating that the current is limited by semi-infinite diffusion of cholesterol on the CNTs electrode.

Five independent measurements were made to assess repeatability and stability of the sensors. The error bars shown in the inset of Figure 6 demonstrate that the sensor has good repeatability and stability of less than 10% under different scan rates.

**Figure 7.**
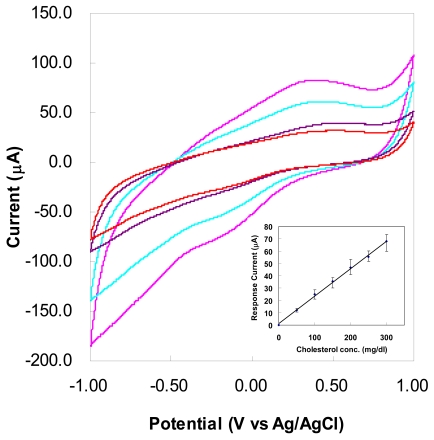
Cyclic voltammograms for cholesterol bioprobe at 100 mV/s scan rate with different cholesterol concentrations. Inset: Amperometric response for bioprobe (oxidation peaks) as a function of cholesterol concentration. The error bars indicates the variation from five independent measurements.

Figure 7 represents the set of waveforms obtained for different concentrations of cholesterol at a specific scan rate (100 mV/s). The difference in the peak current would be the detection capacity for the sensor in a cholesterol concentration at this working electrode surface of 1 mm^2^ active area. It can be seen that the reduction and oxidation peaks become clearly pronounced as the cholesterol concentration increases from 100 to 300 mg/dL. The oxidation and reduction peaks centered around 450 and −250 mV, respectively. In addition, the peaks are more evident and located at higher voltage as scan rate increases at a fixed concentration.

The amperometric response (inset of Figure 7) is the amplitude of oxidation peaks above background from CV curves. An approximately linear relationship between the analyte concentration and the response current of the CNT based electrode can be observed between 50 and 300 mg/dL concentration with a sensitivity of approximately 0.22 μA/mg·dL^−1^, which is considerably higher compared to previously reported CNT bioprobe [8]. The electrochemical sensing performance of the electrode can be attributed to the combination of CNTs properties and efficient enzyme immobilization by two step electrochemical process. Since the response current appeared to be in linear relation with the cholesterol concentration, so the enzyme catalytic reaction of cholesterol is the first-order reaction. The error bars shown in the inset of Figure 7 demonstrate that the sensor has good repeatability and stability of less than 15% over the whole concentration range.

Specificity is one of the most critical issues for biosensors to be used in real environment. To evaluate specificity of the sensors, interferences by four common electroactive species including ascorbic acid, glucose, acetaminophen and uric acid were tested at their normal higher concentration level in serum.

**Figure 8.**
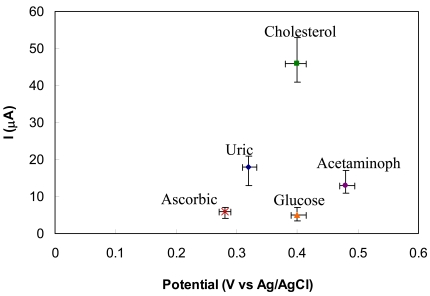
Oxidation peak current vs. corresponding peak potential of cholesterol (200 mg/dL), ascorbic acid (1 mg/dL), glucose (150 mg/dL), acetaminophen (5 mg/dL) and uric acid (7 mg/dL). The error bars indicates the variation for five independent measurements.

Figure 8 demonstrates interference peaks of ascorbic acid (1 mg/dL), glucose (150 mg/dL), acetaminophen and uric acid (7 mg/dL) along with the peak of cholesterol (200 mg/dL). It can be seen that ascorbic acid and glucose give very low interference signals, while acetaminophen and uric acid produce considerable interference. Nevertheless, the interference from these analytes can be minimized by setting the working electrode potential at its peak value (∼0.4 V) since the peaks of interfering analytes locate at different potentials except glucose which has similar peak location but its interference is very low. Finally, long-term stability of the sensor was assessed. The sensors were stored dry at 4 °C and tested every day. It was found that the sensitivity was dropped by 15% after one month due to natural enzyme degradation. Thus, the sensors have a satisfactory life time. Therefore, the present sensor is promising for clinical diagnostics after the calibration to be done with real blood samples.

## 4. Conclusions

In conclusion, electropolymerized vertically aligned carbon nanotubes have been developed to enhance electrochemical sensing of gold coated Si electrode. The fabricated sensors have been characterized for cholesterol detection in the concentration range between 0 and 300 mg/dL cholesterol concentration by standard cyclic voltammogram measurement. An almost linear relationship between the analyte concentration and the response current of the CNT based electrode could be observed between 100 and 300 mg/dL concentration with a high current sensitivity. In addition, good specificity toward glucose, uric acid acetaminophen and ascorbic acid have been obtained. Moreover, sensors have satisfactory stability, repeatability and life time. Thus, the electropolymerized CNT bioprobe can effectively be used for cholesterol detection in normal range of cholesterol concentration in human blood. In addition, this developed scheme has the potentiality to integrate an array of sensors for lab-on-a-chip systems due to its compatibility with the standard Si micro-fabrication technology.
